# Inflammatory-like status and acute stress response in horses after road transport

**DOI:** 10.1038/s41598-023-37069-1

**Published:** 2023-06-17

**Authors:** Francesca Arfuso, Maria Rizzo, Claudia Giannetto, Elisabetta Giudice, Giuseppe Piccione, Francesco Fazio, Roberta Cirincione, Giovanni Cassata, Luca Cicero

**Affiliations:** 1 Department of Veterinary Sciences, University of Messina, 98168 Messina, Italy; 2 Istituto Zooprofilattico Sperimentale della Sicilia “A. Mirri”, 90129 Palermo, Italy

**Keywords:** Physiology, Biochemistry

## Abstract

This study aimed to evaluate the change of white blood cell count, serum concentration of cortisol, C-reactive protein, albumin and globulin fractions in horse after road transport, and to assess the linkage among hypothalamic–pituitary–adrenal axis (HPA) and inflammatory reaction. From 10 horses blood samples were collected at rest, before road transport (218 km) (BT); after unloaded (AT), 30 and 60 min after unloaded (AT30 and AT60) in order to assess white blood cell count (WBC), serum cortisol, C-reactive protein (CRP), total proteins, albumin, α1-, α-2, β1-, β2- and γ-globulins. WBC, cortisol, CRP, α1-, α-2 and β2-globulins values increased after road transport than rest condition (p < 0.001). Albumin and A/G ratio showed lower values after road transport than rest (p < 0.001). Pearson’s test showed a negative correlation between cortisol and the values of WBC, CRP, α1-, α2-, β1-, β2- globulins, and a positive correlation between WBC and serum concentration of CRP, α1- and α2-, β1-, β2-globulins at AT and AT30. The results showed that road transport evokes an inflammatory like-status in horses. Moreover, the activation of HPA and the onset of acute phase reaction in response to road transport seem to be interconnected with effects on horse’s immune status.

## Introduction

Several endogenous and exogenous factors impair the homeostatic equilibrium of organisms inducing stress condition that could affect the welfare status of the animal. Livestock encounters several practices which represent stressors for the animals leading to the modification of normal behavior and growth and to losses of performance^1^. Among these practices, road transport implies several physical and mental stimuli affecting homeostasis and metabolic status in many domestic animal species^2–10^.

Although transport is a common practice in horse management, this experience is known to represent a stressful stimulus for the animal^3,11,12^. Though stress can represent an adaptive process in which physiological and behavioral responses restore an animal back to homeostasis, transport involves the coexistence of multiple stressful conditions including handling, loading, transport itsef, unloading, and, the adaptation to a new environment which could compromise the success of the processes of adaptation to stimuli and, thus, could have negative repercussions on the animal welfare^13^.Though athlete horses are familiarized to travel, after transport, they could show reduced performance than normal^14^. It is recognized that stressors induce the hypothalamic–pituitary–adrenal axis (HPA) initiation as well as the sympathetic nervous system activation; consequently, glucocorticoids and catecholamines are released with consequences on inflammatory status of the animal contributing to an impaired immune function which leads to increased risk of infection and reduced animal welfare^15–20^. Glucocorticoids can inhibit important immune functions such as lymphocyte proliferation^15,16^ and the production of pro-inflammatory cytokines^15^. Catecholamines can exert effects similar to cortisol with lower proliferation^20^ and cytokine production^17,18^. However, they may also lead to immune activation^19,20^. The early response to homeostasis perturbation is known as acute phase response (APR) which is promoted by acute phase proteins (APPs)^21,22^. The APPs are classified in positive APPs, mainly included in α- and β-globulin fractions, whose serum concentration increases significantly in response to inflammation^23^, and in negative APPs, as albumin, showing decreased serum concentration in response to inflammation^24^. In light of the well-known interaction between the endocrine and immune systems under a stressful situation^25^, the evaluation of endocrine markers and immune indices is worthy of investigation for the study of animal’s response to stress and of its capability to restore homeostasis^25^. Though the acute phase protein and stress response of the horses to transport has been previously reported in literature^3,26^, scant data on C-reactive protein change after this condition as well as the relationships between variables measured as part of the acute-phase response are available, so far.

The aims of this study were (1) to evaluate the change of serum concentrations of cortisol, as stress marker, of C-reactive protein, total proteins, albumin, globulin fractions and white blood cell count as inflammation indices in response to road transport in Jumper horses; (2) to assess the possible linkage among HPA, inflammatory and immune reactions in the horses transported by road.

## Material and methods

### Animal and study design

Ten regularly trained Italian Saddle horses (6 geldings, 4 females; 6–10 years old; mean body weight 475 ± 50 kg) were enrolled in the study after the informed consent of the owners. All horses were managed equally at the same horse training center in Messina, Sicily, Italy (latitude 38°10′ 35″ N; longitude 13° 18′ 14″ E), housed in individual boxes (3.5 × 3.5 m), under natural photoperiod and environmental conditions (mean temperature of 26 ± 5 °C and mean relative humidity of 66 ± 4%). All horses were fed 11 ± 2 kg/day/horse of good quality halfa-halfa hay, and 4 ± 0.5 kg/day concentrates (crude protein 16%, crude fat 6%, crude fibre 7.35%, ash 10.09%, sodium 0.46%, lysine 0.85%, methionine 0.35%, omega-3 0.65%), distributed in three meals (at 6:30, 12:00, 19:30), water was available ad libitum. All horses were trained and ridden by the same trainer and rider, respectively. Before starting the study, horses were subjected to clinical examination, routine hematology and biochemistry analyses and all animals were clinically healthy and free from internal and external parasites. The horses took part in an outdoor jumping competition spent in at the “Società Ippica Ragusana” located in Ragusa (latitude 36° 55′ 32″ N; longitude 14° 43′ 27″ E). Therefore, horses were subjected to a road transport from training center (Messina) to the competition’s location through a 10-horse truck. These horses had the same traveling experience, they were well accustomed to traveling in every position and their last travel experience had occurred about 1 month before. Transport was started immediately after loading of the horses. Neither feed nor water were provided during transport, while all horses were fed before transport. The travel start at 09:00am and each animal traveled, tethered with a 50-cm rope on each side of the halter, in an individual tie stall (length 2.0 m; width 0.85 m), giving a total space of about 2 m^2^. The distance covered during travel time was 218 km (about 4 h). The transport led 86 km through cities or through hilly or undulating terrain and 132 km followed highway. Sixty percent of the route was rectilinear, and 40% was characterized by bends, with a minimal road slope of 0.4% and a maximum of 10%. No stops were made during transport. At departure the ambient temperature was of 22.0 °C and the relative humidity was of 50%; at arrival the ambient temperature was of 23.4 °C and the relative humidity was of 54%. Horses were subjected to clinical examination after road transport and no signs of discomfort or pathologies were found. The horses did not show an abnormal excitatory state and they did not show a degree of sweating that would indicate dehydration state.

### Blood sampling and laboratory analysis

From each animal, blood samples were collected by the same operator, before transport (BT), in their stall at 08:30 AM, after being unloaded from the vehicle and housed in the boxes waiting the start of competition (AT); 30 and 60 min after unloaded (AT30 and AT60, respectively). Blood samples were collected by jugular venipuncture into 2-mL vacutainer tubes containing ethylenediaminetetraacetic acid (EDTA), and into 8-mL vacutainer tubes with cloth activator (Terumo Co., Tokyo, Japan). As previously described^10,27^, immediately after collection, blood samples were placed in refrigerated bags and transported to the laboratory for the analysis. The EDTA whole blood samples were processed in the laboratory within 2 h for the evaluation of WBC count and hematocrit values that was performed by means of an automated hematology analyzer (HeCoVet C; SEAC, Florence, Italy). The blood samples collected into vacuum tubes containing clot activator were allowed to clot for 20 min at the room temperature prior to centrifugation at 1300×*g* for 10 min and the obtained sera were stored at − 20 °C until analysis^10^. On obtained serum samples the concentration of C-reactive protein (CRP), cortisol, total proteins and globulin fractions was assessed. The serum concentrations of CRP and cortisol were evaluated using enzyme-linked immunosorbent assay (ELISA) kits specific for equine species (cortisol Horse ELISA kit, Abnova, Walnut, CA; CRP Horse ELISA kit, ab190527, Abcam, Boston, USA) by means of a micro-well plate reader (Sirio, SEAC, Florence, Italy). All calibrators and samples were run in duplicate and samples exhibited parallel displacement to the standard curve for each ELISA analysis^10^. The sensitivity of the CRP kit was 1.198 ng/mL, whereas both the intra- and the inter-assay coefficients of variation were at < 10%. The sensitivity of the cortisol kit was 1 ng/mL, whereas the intra-assay and inter-assay of variation were 6% and 6.8%, respectively. Serum total protein concentration was measured by means of automated UV spectrophotometer (Slim, SEAC, Florence, Italy) using the Biuret method with commercially available kit (Biosystems S.A., Barcelona, Spain), and the bovine albumin (6.02 g/dL) as standard protein (Biosystems S.A., Barcelona, Spain). Electrophoresis for protein fraction assessment was performed using an automated system (Selvet24, Seleo Engineering, Naples, Italy) according to the procedures suggested by the manufacturer and as previously described^27^. All samples were analyzed by the same operator, who determined the lines separating fractions in the densimeter tracing^10^. The major protein fractions were divided into albumin, α1-, α-2, β1-, β2- and γ-globulins, from the cathode to the anode, according to the recommendation by the manufacturer. Relative protein concentrations within each fraction were determined as the optical absorbance percentage; then the absolute concentration (g/dL) and albumin/globulin ratio (A/G) were calculated using the total protein concentration^10^.

### Statistical analysis

Data were tested for normality using the Shapiro–Wilk test and resulted normally distributed (p > 0.05). One-way analysis of variance for repeated measures was applied to assess significant effect of road transport on WBC and hematocrit values and on serum concentration of CRP, cortisol, total proteins, albumin and globulin fractions (α1-, α-2, β1-, β2-, γ-globulins). When significant differences were found, Bonferroni post hoc comparison was applied. Pearson's correlation coefficients were computed to evaluate the possible correlation between the serum concentration of cortisol and the serum values of acute phase proteins (i.e. CRP, albumin, α1-, α-2, β1-, β2-globulins) and the values of WBC obtained in horses at each sampling time. To confirm these relationships and to determine the degree of correlations, a linear regression model (y = a + bx) was applied. p values < 0.05 were considered statistically significant. The statistical analysis was performed using the software Prism v. 9.00 (Graphpad Software Ldt., USA, 2020).

### Ethical approval

All treatments and animal care reported previously were carried out following the standards recommended by the European Directive 2010/63/EU for animal experiments. All animals were enrolled in the study after the written consent of the owners in compliance with the Italian Regulation D.L. 116/1992. The animal study was reviewed and approved by Ethics Committee of the Department of Veterinary Sciences, University of Messina.

## Results

**Figure 1:**
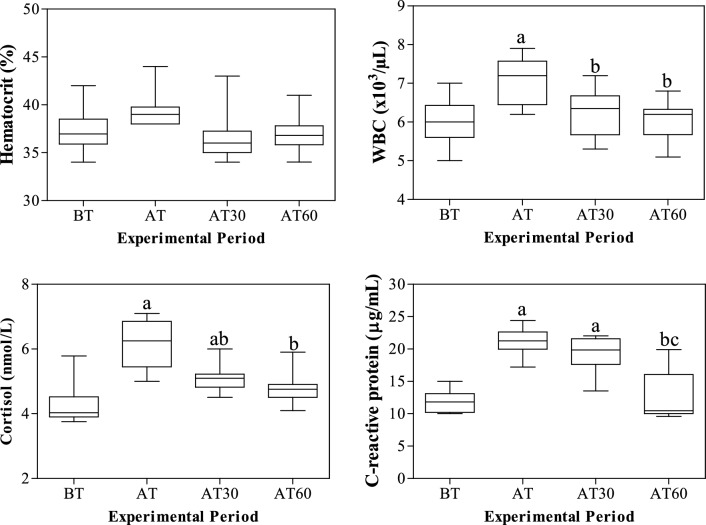
Mean values ± standard error of the mean (± SEM) of hematocrit, white blood cell (WBC), C-reactive protein and cortisol obtained from horses before transport (BT) in their stall at 08:30 AM, after being unloaded from the vehicle and housed in the boxes waiting the start of competition (AT), 30 and 60 min after being unloaded from the vehicle (AT30 and AT60, respectively). Significant effect of road transport (P<0.001): ^a^vs BT; ^b^vs AT; ^c^vs AT30.

**Figure 2:**
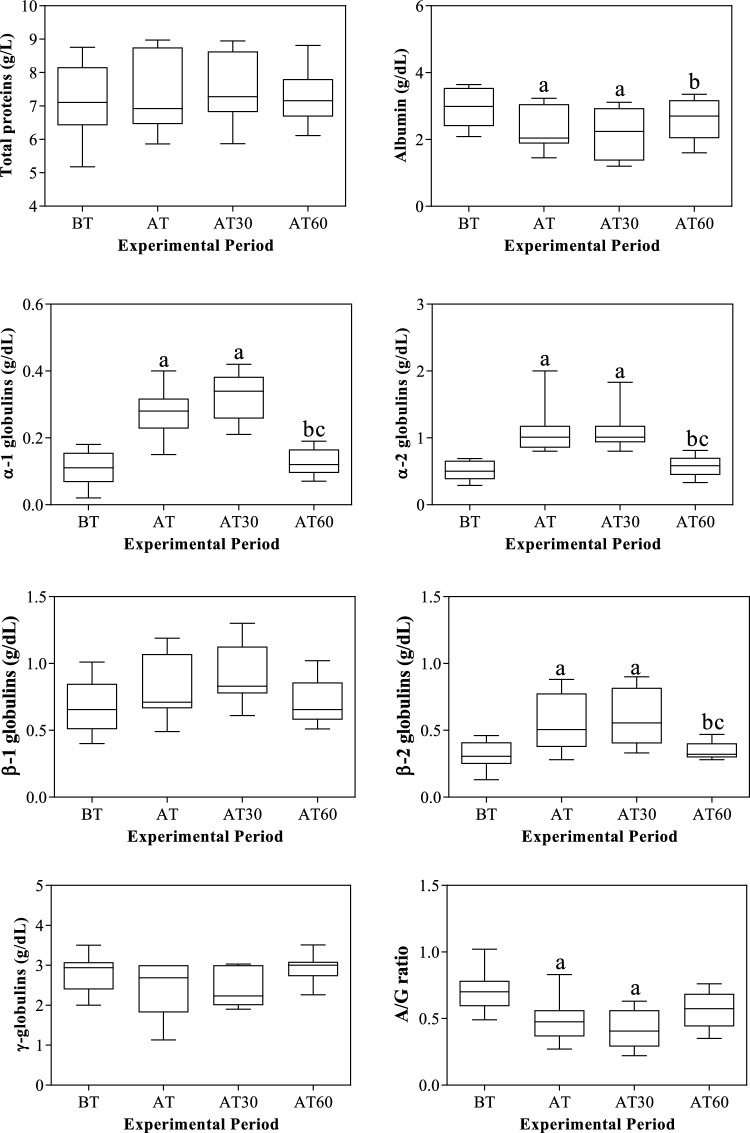
Mean values ± standard error of the mean (± SEM) of serum total proteins, albumin, globulin fractions (i.e. α1-, α-2, β1-, β2- and γ-globulins) and albumin/globulin ratio (A/G) obtained from horses before transport (BT) in their stall at 08:30 AM, after being unloaded from the vehicle and housed in the boxes waiting the start of competition (AT), 30 and 60 min after being unloaded from the vehicle (AT30 and AT60, respectively). Significant effect of road transport (P<0.001): ^a^vs BT; ^b^vs AT; ^c^vs AT30.

**Figure 3:**
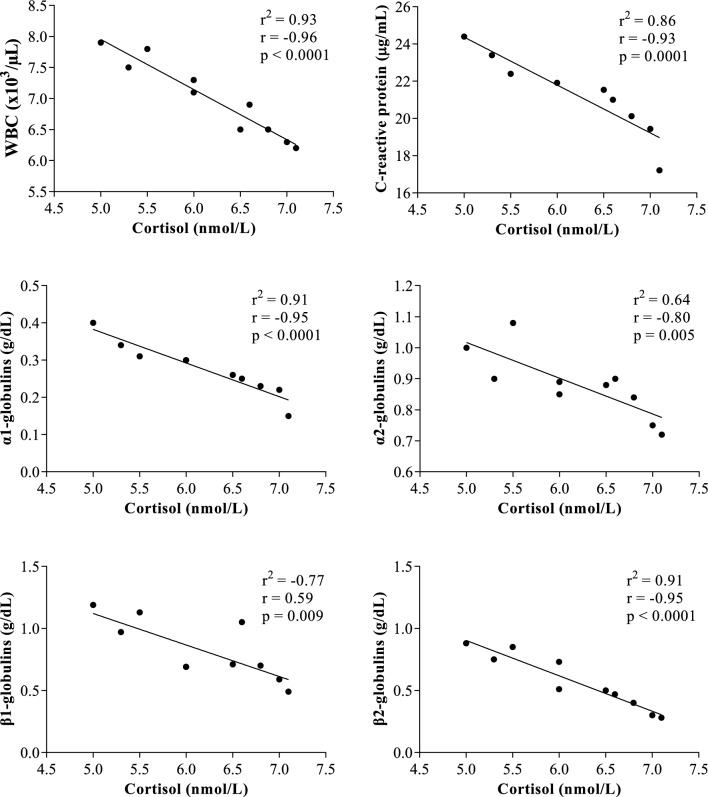
Statistically significant linear regression results obtained between the serum concentration of cortisol and the values of white blood cell (WBC), C-reactive protein, α1-, α-2, β1- and β2-globulins recorded from horses after being unloaded from the vehicle and housed in the boxes waiting the start of competition (AT).

**Figure 4:**
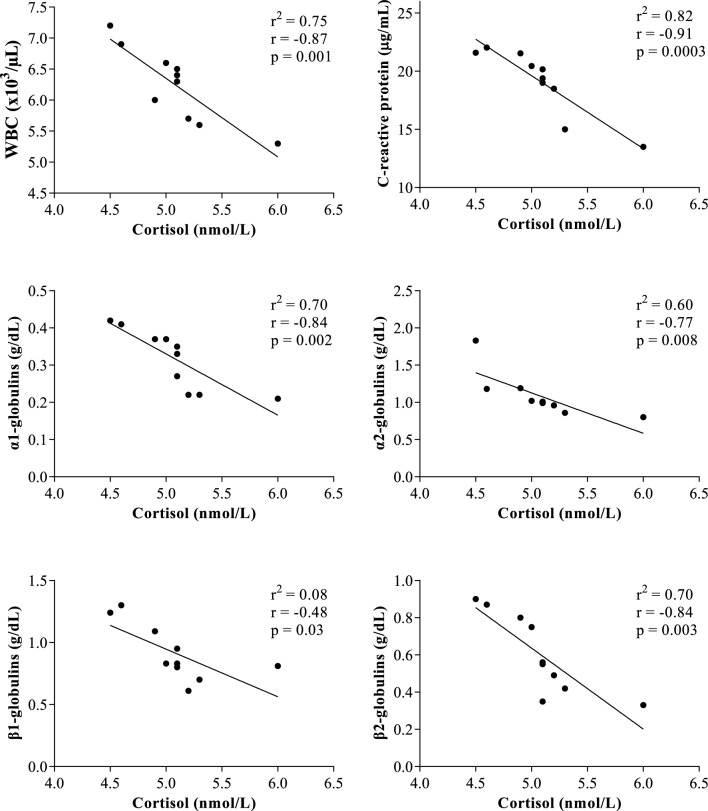
Statistically significant linear regression results obtained between the serum concentration of cortisol and the values of white blood cell (WBC), C-reactive protein, α1-, α-2, β1- and β2-globulins recorded from horses 30 min after being unloaded from the vehicle (AT30).

**Figure 5:**
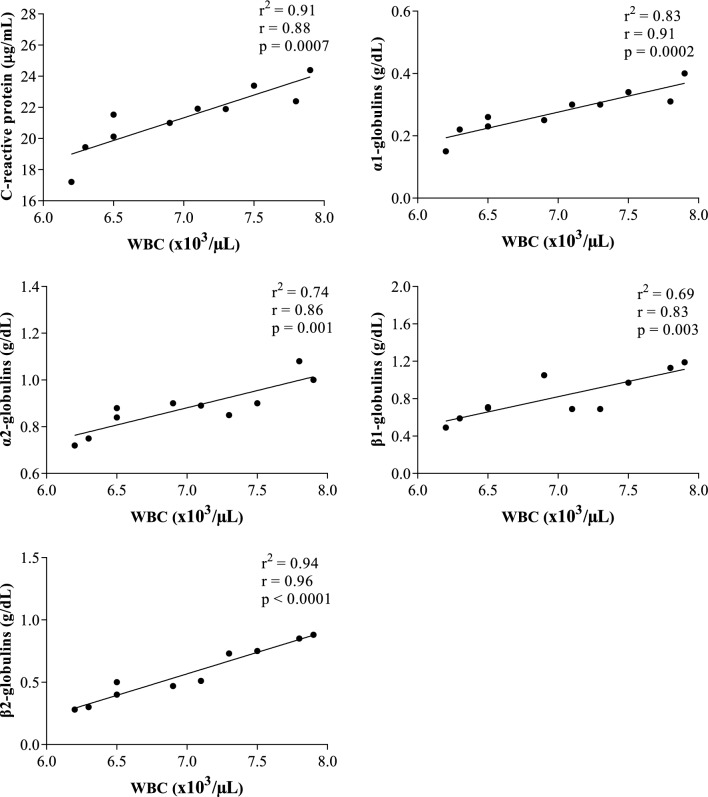
Statistically significant linear regression results obtained between the values of white blood cell (WBC) and the serum concentrations of cortisol, C-reactive protein, α1-, α-2, β1- and β2-globulins recorded from horses after being unloaded from the vehicle and housed in the boxes waiting the start of competition (AT).

**Figure 6:**
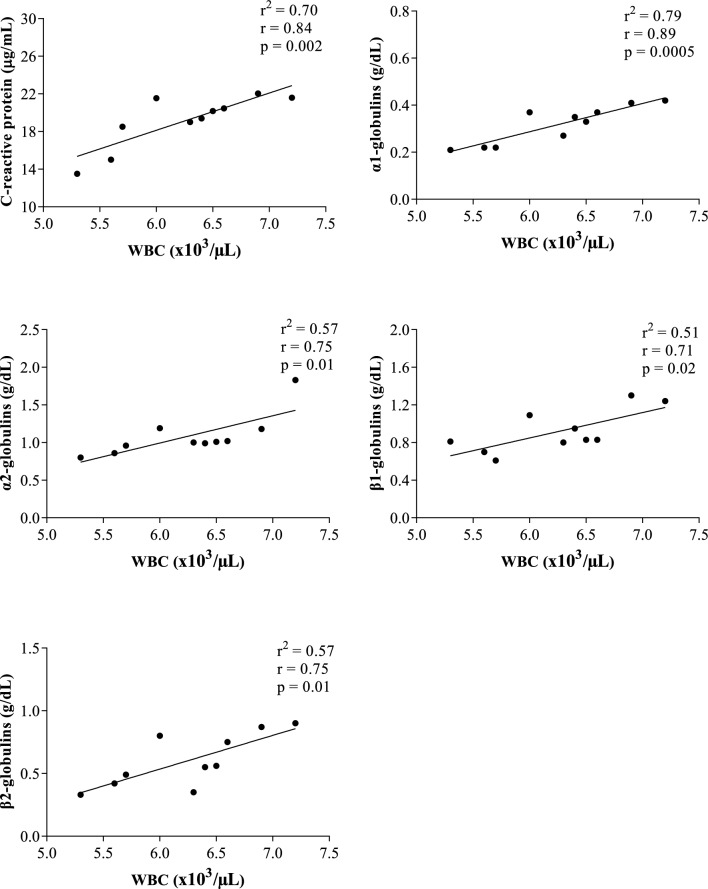
Statistically significant linear regression results obtained between the values of white blood cell (WBC) and the serum concentrations of cortisol, C-reactive protein, α1-, α-2, β1- and β2-globulins recorded from horses 30 min after being unloaded from the vehicle (AT30).

All results were expressed as mean ± standard error of the mean (SEM). The application of one-way ANOVA revealed no statistical significances on the serum total proteins, β1- and γ-globulin fractions (p > 0.05). As showed in Fig. 1, higher haematocrit values were observed in horses after being unloaded from the vehicle and housed in the transit stalls (AT, 38.7 ± 0.21) than the other time points of monitoring period (BT, 37.4 ± 0.73%; AT30, 36.7 ± 0.79%; AT60, 37.0 ± 0.61%); however, these changes were not statistically significant (p > 0.05). Contrariwise, a statistically significant effect of transport (p < 0.001) on WBC and on serum concentration of cortisol, CRP, albumin, α1-, α2-, β2-globulins and A/G ratio was found. Specifically, the WBC values and the serum concentrations of cortisol, CRP, α1-, α2- and β2-globulins statistically increase after road transport compared with the values recorded at rest condition (p < 0.001, Figs. 1, 2), albumin and A/G ratio showed statistically significant lower values after road transport than rest (p < 0.001, Fig. 2). According to correlation results, the serum concentration of cortisol showed a significant negative correlation with WBC, CRP, α1-, α2-, β1- and β2- globulin fractions immediately after the end of road transport (AT) and after 30 min from the end of road transport (AT30), whereas no significant correlation among these parameters was found at rest condition and after 60 min from the end of road transport (Table 1). The WBC values were positively correlated with CRP, α1-, α2-, β1- and β-2globulin fractions immediately after the end of road transport (AT) and after 30 min from the end of road transport (AT30), whereas no significant correlation among these parameters was found at rest condition and after 60 min from the end of road transport (Table 1). The significant outcomes of Pearson’s correlation were confirmed by the linear regression model results (Figs. 3, 4, 5, 6).

**Table 1:** Coefficients of correlation (Pearson’s r and p values) among the values of serum cortisol and/or white blood cell (WBC) and the serum levels of C-reactive protein, albumin and globulin fractions (i.e. α1-, α-2, β1-, β2- and γ-globulins) obtained from horses before transport (BT) in their stall at 08:30 AM, after being unloaded from the vehicle and housed in the transit stalls (AT), 30 and 60 min after unloaded (AT30 and AT60, respectively).

	Cortisol (nmol/L)	WBC (× 10^3^/μL)	C-reactive protein (µg/mL)	Albumin (g/dL)	α1-Globulins (g/dL)	α2-Globulins (g/dL)	β1-Globulins (g/dL)	β2-Globulins (g/dL)	γ-Globulins (g/dL)
BT									
Cortisol (nmol/L)		r = **− **0.06 p = 0.87	r = 0.32 p = 0.36	r = 0.06 p = 0.86	r = 0.47 p = 0.17	r = 0.23 p = 0.51	r = **− **0.36 p = 0.31	r = 0.14 p = 0.71	r = 0.18 P = 0.62
WBC (× 10^3^/μL)	r = **− **0.06 p = 0.87		r = **− **0.09 p = 0.80	r = 0.21 p = 0.55	r = **− **0.35 p = 0.32	r = **− **0.44 p = 0.20	r = 0.11 p = 0.75	r = **− **0.03 p = 0.94	r = 0.17 P = 0.63
AT									
Cortisol (nmol/L)		**r = − 0.96** **p < 0.0001**	**r = − 0.93** **p = 0.0001**	r = **− **0.37 p = 0.30	**r = − 0.95** **p < 0.0001**	**r = − 0.80** **p = 0.005**	**r = − 0.77** **p = 0.009**	**r = − 0.95** **p < 0.0001**	r = **− **0.39 P = 0.27
WBC (× 10^3^/μL)	**r = − 0.96** **p < 0.0001**		**r = 0.88** **p = 0.007**	r = 0.48 p = 0.16	**r = 0.91** **p = 0.002**	**r = 0.86** **p = 0.001**	**r = 0.83** **p = 0.003**	**r = 0.96** **p < 0.0001**	r = 0.34 P = 0.33
AT30									
Cortisol (nmol/L)		**r = − 0.86** **p = 0.001**	**r = − 0.91** **p = 0.0003**	r = **− **0.54 p = 0.11	**r = − 0.84** **p = 0.003**	**r = − 0.77** **p = 0.009**	**r = − 0.64** **p = 0.03**	**r = − 0.84** **p = 0.003**	r = 0.22 P = 0.54
WBC (× 10^3^/μL)	**r = − 0.86** **p = 0.001**		**r = 0.84** **p = 0.003**	r = 0.56 p = 0.09	**r = 0.89** **p = 0.0005**	**r = 0.75** **p = 0.01**	**r = 0.71** **p = 0.02**	**r = 0.75** **p = 0.01**	r = **− **0.27 P = 0.44
AT60									
Cortisol (nmol/L)		r = **− **0.36 p = 0.30	r = 0.35 p = 0.32	r = 0.12 p = 0.73	r = 0.05 p = 0.88	r = 0.33 p = 0.34	r = **− **0.15 p = 0.68	r = 0.15 p = 0.68	r = **− **0.07 P = 0.84
WBC (× 10^3^/μL)	r = **− **0.36 p = 0.30		r = **− **0.18 p = 0.62	r = **− **0.09 p = 0.81	r = **− **0.11 p = 0.76	r = 0.32 p = 0.36	r = **− **0.15 p = 0.68	r = **− **0.19 p = 0.59	r = 0.09 P = 0.80

Values of p < 0.05 were considered statistically significant, and highlighted in bold.

## Discussion

Horses are among the most transported animals in Europe^28^. Being considered one of the most stressful events in an animal’s life, transport represents a major welfare concern^29^. The most stressful stages during transport is represented by the loading phase in livestock^9,30^ and in sport horses^10^. The body’s early defense in response to homeostasis perturbation due to trauma, inflammation and/or stressors exposure is known as APR. The analysis of the results obtained in the current study showed an immune, inflammatory and stress reactions of horses in response to road transport. Specifically, the concentrations of WBC, CRP, α1-, α-2 and β2-globulins showed an increasing trend up to 30 min after the end of the transport followed by a subsequent decrease after 60 min from the end of road transport, whereas albumin showed an opposite trend, reflecting and APR. The changes of the inflammation markers herein found agree with previous studies investigating the effect of road transport on inflammation status of cattle, horses and ewes^2,3,5^. Several published papers with conflicting and contradictory statements regarding transport of horses are available in scientific literature. A study carried out to verify whether the road transport of horses over distances of 50 and 300 km induces changes in the values of acute phase proteins showed that the distances covered with the horses did not affect the serum concentration of acute phase proteins^31^. Contrariwise, another study showed that long distance transportation was associated with an acute phase response characterized by neutrophilia, hyperglobulinemia, and an impairment of the immune system evidenced by reduced lymphocyte responsiveness^32^. A study carried out on pigs showed that several factors as ambient temperatures above 22 °C, distance traveled 26 km or above, travel duration between 38 and 66 min, affect animal welfare as well as meat quality^33,34^.

The APPs have both bacteriostatic and immune-modulatory effects^24^, therefore the increase in the studied APPs could be connected to an initial changes of immune system’s response of the transported horses as highlighted by the changes in WBC herein found and by the positive correlation found between the values of WBC and the serum concentration of CRP, α1-, α-2, β1- and β2-globulins after the end of road transport (AT) and after 30 min from the end of road transport (AT30). The changes of WBC values found in the current study in response to stress related to road transport agree with the results of previous investigation carried out on horses, bulls and goats^35–38^. The changes of WBC values found in the current study in response to stress related to road transport agree with the results of previous investigation^36^, and these modifications have been associated with an increase in neutrophils and a decrease in lymphocytes^37,38^. Noteworthy, the variations in APPs and WBC values could be the consequence of increased cortisol release. As a matter of facts, according to the results gathered in the current study, the serum cortisol concentration followed the same trend shown by WBC and inflammation indices with increased values after road transport. The increased serum levels of cortisol found in investigated horse after road transport agree with previous findings gathered in a study carried out on Thoroughbred horses transported by road^10^ and strengthen the evidence that the mental and the physical pressure during transport represents an acute stress for animal resulting in the activation of HPA axis and in the rise of serum cortisol concentration^25^. In addition to being known as an acute stress hormone, cortisol is known as a metabolic driver and modulator of the animal's immune and inflammatory responses. Specifically, it has been suggested that, during stress response, cortisol plays a modulatory action on immune status of the animal actin on down-regulation of gene intricate in T-cell activation as well as on inflammatory state moderating interleukins production and exhibiting a stimulatory effect on hepatic APP synthesis^24^.

This suggestion is emphasized by the negative relationship observed between the serum cortisol concentration and the levels of most of the immune and inflammatory indices herein investigated (i.e. WBC CRP, α1-, α2-, β1- and β2-globulins). The negative correlation found between acute stress hormone and the immune and inflammatory parameters seems to suggest an inter-communication among HPA axis, immune and inflammatory response during the stress condition as transport. It is well known that inflammatory response is beneficial as it attracts circulating immune effector cell to face infection, however, an excessive inflammatory response can injure tissues and organs. A tight control of the expression of inflammatory and pro-inflammatory mediators during an inflammatory response is needed in order to avoid animal health impairment. Therefore, according to the results gathered in the current study, it could be speculated that, similarly to what happen during physiological stress response as physical exercise, increased cortisol concentration counteracts the excessive inflammatory responses during stress condition as road transport averting tissues injury.

## Conclusion

The current study showed the evidence that road transport evokes an acute stress response as well as an inflammatory like-state in horses as highlighted by the increased levels of cortisol, WBC, CRP, α- and β-globulin fractions and the decreased levels of albumin after transport compared with the values recorded at pre-transport time. Moreover, the HPA axis activation and the APR onset in response to a stressful stimulus such as road transport seem to be interconnected with possible effects on the immune status of the horse. This study suggests that the evaluation of cortisol together with CRP and the other non-specific markers of inflammation including the serum protein fractions, could be considered as a useful tool in defining the objective health status in horses after transport and, therefore, to gain useful information to safeguard the animal’s welfare.

## Data availability

The datasets used and/or analysed during the current study are available from the corresponding author on reasonable request.
